# The Effects of *Bifidobacterium breve* on Immune Mediators and Proteome of HT29 Cells Monolayers

**DOI:** 10.1155/2015/479140

**Published:** 2015-02-22

**Authors:** Borja Sánchez, Irene González-Rodríguez, Silvia Arboleya, Patricia López, Ana Suárez, Patricia Ruas-Madiedo, Abelardo Margolles, Miguel Gueimonde

**Affiliations:** ^1^Department of Microbiology and Biochemistry of Dairy Products, Instituto de Productos Lácteos de Asturias, Consejo Superior de Investigaciones Científicas (IPLA-CSIC), Paseo Río Linares s/n, Villaviciosa, 33300 Asturias, Spain; ^2^Department of Functional Biology, Immunology Area, University of Oviedo, Oviedo, 33006 Asturias, Spain

## Abstract

The use of beneficial microorganisms, the so-called probiotics, to improve human health is gaining popularity. However, not all of the probiotic strains trigger the same responses and they differ in their interaction with the host. In spite of the limited knowledge on mechanisms of action some of the probiotic effects seem to be exerted through maintenance of the gastrointestinal barrier function and modulation of the immune system. In the present work, we have addressed *in vitro* the response of the intestinal epithelial cell line HT29 to the strain *Bifidobacterium breve* IPLA20004. In the array of 84 genes involved in inflammation tested, the expression of 12 was modified by the bifidobacteria. The genes of chemokine CXCL6, the chemokine receptor CCR7, and, specially, the complement component C3 were upregulated. Indeed, HT29 cells cocultivated with *B. breve* produced significantly higher levels of protein C3a. The proteome of HT29 cells showed increased levels of cytokeratin-8 in the presence of *B. breve*. Altogether, it seems that *B. breve* IPLA20004 could favor the recruitment of innate immune cells to the mucosa reinforcing, as well as the physical barrier of the intestinal epithelium.

## 1. Introduction

Probiotics are live microorganisms which when administered in adequate amounts confer a health benefit on the host [1], the genus* Bifidobacterium* being among the most widely used. These microorganisms are common members of the human gut microbiota and they predominate in breast-fed infants [2]. Several beneficial health effects have been attributed to specific probiotic strains [3]. Although the knowledge on probiotic mechanisms of action is still limited some of these beneficial effects are exerted through their role in the maintenance of the gastrointestinal barrier function and by modulating the immune system [4, 5].

The interest in the immunomodulatory properties of probiotic bacteria derives from the observations that intestinal microbiota plays a critical role in the development and regulation of the immune system [6]. It is known that different probiotic bacteria present different effects upon the immune system [7, 8], making necessary the characterization of the effects of each specific potentially probiotic strain. Some strains promote Th1 responses, characterized by the production of IFN*γ* and TNF*α*, whereas other strains induce anti-inflammatory cytokines generating a Th2 profile [7, 8]. To determine these properties, the direct effect of the interaction of probiotic bifidobacteria with immune cells, either total peripheral blood mononuclear cells (PBMCs) or isolated immune cell types, is often studied. However, the potential effect of the cross talk between bifidobacteria and epithelial cells upon the immune system has received less attention. The intestinal epithelium separates microorganisms from the underlying immune cells. It consists of a layer of cells, mainly enterocytes, and a mucus layer that coats the epithelium [9]. Moreover, different immune cells are localized in the gut associated lymphoid tissue, which constitute the first contact point between gut commensals and the immune system [10]. Consequently, assessing the effect of the interaction of potentially probiotic* Bifidobacterium* strains with the gut mucosa constitutes an important task for both probiotics selection and understanding of their mechanisms of action. This understanding would allow selection of specific strains with the desired properties for a specific application.

Previous studies carried out on the breast-milk isolate* Bifidobacterium breve* IPLA20004 [11] by our group indicated the ability of this strain to induce Th1 polarization of lymphocytes and to increase the physical resistance of the intestinal mucosa [12, 13]. These results suggest that this strain may be of interest for increasing the intestinal barrier against pathogens, firstly by strengthening the physical resistance of the epithelial layer and secondly by modulating the immune system towards a preactivated steady state. Moreover, some effects of the strain on the expression of chemokines and their receptors have been previously suggested [13]. To this regard an effect on the production of chemokines by intestinal epithelial cells may have a direct impact on the immune system by affecting the recruitment of immune cells to the mucosa.

For the above-mentioned reasons we decided to evaluate the effect of* B. breve* IPLA20004 on the expression of genes related to the inflammatory response and on the production of cytokines, by the human intestinal epithelial cell line HT29. Moreover, the effect of the strain on HT29 cells was also assessed by proteomic analyses.

## 2. Materials and Methods

### 2.1. Bacteria Culture Conditions

To evaluate the effects of the* B. breve* IPLA20004 on HT29 cells, cultures were freshly prepared by growing the microorganisms in MRS medium (Difco, Becton, Dickinson and Company, Le Pont de Claix, France) supplemented with a 0.25% L-cysteine (Sigma Chemical Co., St. Louis, MO, USA) (MRSc) at 37°C under anaerobic conditions (10% H_2_, 10% CO_2_, and 80% N_2_) in a chamber Mac 500 (Don Whitley Scientific, West Yorkshire, UK).

### 2.2. HT29 Cell Line Culture Conditions

The epithelial intestinal cell line HT29 (ECACC number 91072201), derived from human colon adenocarcinoma, was purchased from the European Collection of Cell Cultures (Salisbury, UK). HT29 cell culture passages 146-147 were used for the experiments. The cell line was maintained in McCoy's medium supplemented with 3 mM L-glutamine, 10% (v/v) heat-inactivated bovine fetal serum, and a mixture of antibiotics to give a final concentration of 50 *μ*g/mL penicillin, 50 *μ*g/mL streptomycin, 50 *μ*g/mL gentamicin, and 1.25 *μ*g/mL amphotericin B. All media and supplements were obtained from Sigma. The incubations took place at 37°C, 5% CO_2_ in an SL water-jacketed CO_2_ incubator (Sheldon Mfg. Inc., Cornelius, Oregon, USA). Culture media were changed every two days and the cell line was trypsinized with 0.25% trypsin-EDTA solution (Sigma) following standard procedures. For gene expression experiments and protein profile determinations, 10^5^ cells/mL were seeded in 24-well plates and incubated to reach a confluent and differential state (reaching about 10^7^ HT29 cells/mL) after 13 ± 1 days.

### 2.3. Gene Expression Analysis

*B. breve* IPLA20004 was grown overnight in MRSc, harvested by centrifugation, washed twice with Dulbecco's PBS buffer (Sigma), and resuspended in McCoy's medium without antibiotics. Five hundred *μ*L of a bacterial suspension containing 10^8^ cfu/mL (as determined by plate counting) in McCoy's medium or McCoy's medium without bacteria (control) was added to each well containing HT29 monolayers (bacteria/HT29 cell ratio 10 : 1) previously washed twice with Dulbecco's PBS to remove the antibiotics. Plates were then incubated for 6 h at 37°C, 5% CO_2_ in a Heracell 240 incubator (Thermo Electron LDD GmbH, Langenselbold, Germany). After incubation the culture media were removed and stored at −80°C, the monolayers were resuspended in 500 *μ*L of RNA Protect Cell Reagent (Qiagen GmbH, Hilden, Germany), and the cells were kept frozen at −80°C until RNA extraction. At least three independent experiments were carried out.

RNA from HT29 cells was extracted by using the RNeasy Plus Mini Kit (Qiagen) and QIAshredder homogenizer columns (Qiagen) following manufacturer instructions. Quality of RNA was monitored by gel electrophoresis and it was quantified by using an Epoch apparatus (BioTek Instruments, Inc., Winooski, VT, USA). For reverse-transcriptase PCR analyses 1 *μ*g of RNA was reverse-transcribed to cDNA by using the RT2 First Strand Kit (SABiosciences, Qiagen, Frederick, MD, USA), and gene expression was quantified by using the 96-well RT2 Profiler PCR Array for human inflammatory cytokines and receptors (SABiosciences) following manufacturer's instructions. The array comprises 84 key genes involved in the inflammatory response including chemokine and cytokine genes (CCL1 [I-309], CCL11 [eotaxin], CCL13 [mcp-4], CCL15 [MIP-1d], CCL16 [HCC-4], CCL17 [TARC], CCL18 [PARC], CCL19, CCL2 [mcp-1], CCL20 [MIP-3a], CCL21 [MIP-2], CCL23 [MPIF-1], CCL24 [MPIF-2/eotaxin-2], CCL25 [TECK], CCL26, CCL3 [MIP-1a], CCL4 [MIP-1b], CCL5 [RANTES], CCL7 [mcp-3], CCL8 [mcp-2], CXCL1, CXCL10 [IP-10], CXCL11 [I-TAC/IP-9], CXCL12 [SDF1], CXCL13, CXCL14, CXCL2, CXCL3, CXCL5 [ENA-78/LIX], CXCL6 [GCP-2], CXCL9, IL13, IL8, IFNA2, IL10, IL13, IL17C, IL1A, IL1B, IL1F10, IL1F5, IL1F6, IL1F7, IL1F8, IL1F9, IL22, IL5, IL8, IL9, LTA, LTB, MIF, SCYE1, SPP1, and TNF), chemokine and cytokine receptor genes (CCR1, CCR2, CCR3, CCR4, CCR5, CCR6, CCR7, CCR8, CCR9, CX3CR1, XCR1 [CCXCR1], IL1R1, IL1RN, IL5RA, IL8RA, IL8RB, IL9R, IL10RA, IL10RB, and IL13RA1), other genes involved in the inflammatory response (ABCF1, BCL6, C3, C4A, C5, CEBPB, CRP, ICEBERG, LTB4R, and TOLLIP), and five housekeeping genes (B2 M, HPRT1, RPL13A, GAPDH, and ACTB) for normalization of data.

### 2.4. Cytokines and C3a Determination

Cytokine and C3a levels in the cell culture supernatants of HT29 cells cultured with or without* B. breve* as indicated above were quantified by using the High Sensitivity ELISA Kits for human IL10, IL12p70, IL1*β*, and TNF*α* and the Platinum ELISA Kits for human IL8 and C3a (eBioscience Inc., San Diego, CA, USA). Colour development after ELISA was measured in a Modulus Microplate Photometer (Turner Biosystems, Sunnyvale, CA, USA). All the results were expressed as pg/mL. Detection limits for the ELISA kits used were 0.05, 0.1, 0.05, 0.13, 2, and 70 pg/mL for IL10, IL12p70, IL1*β*, TNF*α*, IL8, and C3a, respectively.

### 2.5. Determination of the Proteomic Profiles

*B. breve* IPLA20004 was grown and added to the wells containing HT29 as previously indicated. Plates were then incubated for 3 h at 37°C, 5% CO_2_, gently washed three times with Dulbecco's PBS buffer to remove the nonadhered bacteria, and the HT29 monolayers were kept for further proteomic analysis.

For protein extraction and two-dimensional electrophoresis analysis, HT29 monolayers were disaggregated with 440 *μ*L of lysis buffer (30 mM Tris, 7 M urea, 2 M thiourea, 4% (w/v) CHAPS, and 100 mM DTT; all reagents were purchased by GE Healthcare Life Sciences) containing complete protease inhibitors (Roche Diagnostics, Mannheim, Germany). Total protein from the cell suspensions was obtained by sonication for one min in ice-chilled water (two cycles), with one min of delay between the two cycles. After adding 2 mg of RNase A (Sigma-Aldrich) and 100 U of DNase I (Sigma-Aldrich), the cell lysates were incubated for 30 min at RT. Finally, the pellet was centrifuged for 10 min at 16,000 g and 4°C to precipitate insoluble components and cell debris. Protein concentration was estimated using the BCA Protein Assay Kit (Pierce, Rockford, IL).

Isoelectric focusing (IEF) was performed in immobilized pH gradient (IPG) strips containing a nonlinear pH range of 3–10 (GE Healthcare Life Sciences), using 500 *μ*g of protein. When needed, lysis buffer was added up to 450 *μ*L. In all the cases, the IPG-buffer corresponding to pHs 3–10 was added to a final concentration of 0.5% (v/v). IEF was conducted at 20°C for 60,000 Vhrs in an IPGphor system (GE Healthcare Life Sciences). Proteins were resolved by SDS-PAGE (12.5% w/v polyacrylamide gel) and stained with GelCode Blue Safe Protein Stain (Pierce). Gels were scanned using ImageScanner (GE Healthcare Life Sciences), and spot detection and volume quantification were performed with ImageMaster Platinum software (version 5.00, GE Healthcare). The relative volume of each spot was obtained by determining the spot intensity in pixel units and normalizing that value to the sum of the intensities of all the spots of the gel. Each experiment was performed independently four times, and the differences in normalized volumes were analyzed statistically using paired Student's *t*-tests (control condition versus presence of the bifidobacteria strain).

### 2.6. Statistical Analyses

Differences in the measured variables, between the control HT29 cells and those exposed to the* B. breve* strain, were evaluated by one-way ANOVA test. Results were represented by mean ± standard deviation. The SPSS 18.0 statistical software package (SPSS Inc., Chicago, IL, USA) was used for all determinations and a value of *P* < 0.05 was considered significant.

## 3. Results

### 3.1. Effect of* B. breve* IPLA20004 on the Expression of Genes Mediating the Inflammatory Response in HT29 Cells

When using the human inflammatory cytokines and receptors pathway focused RT-PCR array, comprising 84 key genes involved in the inflammatory response, we observed some statistically significant changes in gene expression in HT29 cells after coincubation with* B. breve* IPLA20004. These changes were in general modest and most of the studied genes were expressed at low basal levels (Ct values around 30, data not shown), with the exception of CCL25 (Ct value of 19 in the control HT29 cells) and CCR1 (Ct value 26 in the control). The genes whose expression was significantly modified by the strain are shown in Table 1. The expression of chemokine genes CCL2, CCL25, and CXCL14 and the cytokines genes IL10 and IL13 genes was significantly downregulated. On the contrary the gene for CXCL6 chemokine was found to be upregulated. With regard to chemokine receptor genes, a statistically significant downregulation of CCR1, CCR4, CCR5, and XCR1 and induction of CCR7 were observed. Interestingly,* B. breve* IPLA20004 upregulated very significantly (17-fold) the expression of the complement component C3 (Table 1). No statistically significant differences were observed for any of the other genes analyzed in the RT-PCR array (data not shown).

### 3.2. Effect of* B. breve* IPLA20004 on Cytokines and C3 Production by HT29 Cells

The levels of the different cytokines measured, as well as those of C3a, in supernatants of HT29 cells are shown in Table 2. In general the levels detected were low, in some cases being barely over the detection limits of the ELISA kits used. No statistically significant differences between control and* B. breve*-exposed HT29 cells were observed for IL10, IL1*β*, TNF*α*, or IL8 levels. On the contrary coculture of HT29 cells with* B. breve* IPLA20004 significantly increased the production of IL12p70 and C3a, although for the former cytokine the detected levels (0.19 and 0.3 pg/mL for control and* B. breve*-exposed HT29 cells, resp.) were only slightly above the detection limit of the technique used and, therefore, the relevance of this observation is unclear.

### 3.3. Effect of* B. breve* IPLA20004 on the Proteome of HT29 Cells

The comparison of the proteomes of HT29 cells cocultured with or without* B. breve* revealed that two proteins were significantly (*P* < 0.05) upregulated in the HT29 cells by the strain* B. breve* IPLA20004 (see Figure 1 in the Supplementary Material available online at http://dx.doi.org/10.1155/2014/479140). These proteins were excised from the gels and identified as cytokeratin-8 (2.8 times fold induction) and the chain A of the tapasin-ERp57 (4.7 times fold induction).

## 4. Discussion

The interaction of bacteria with intestinal epithelial cells may play a role in immune modulation by modifying gene expression and local immune environment through, for instance, production of chemokines and other immune active molecules. Chemokines are chemotactic cytokines that guide the migration of cells regulating leukocyte traffic and exert their effects by interacting with their specific receptors that are selectively found on the surfaces of their target cells.

We studied the interaction of* B. breve* IPLA20004 with colonic epithelial cells HT29 and found changes in the expression of genes related to the inflammatory response and immune cell chemotaxis in the HT29 cell line. The strain was observed to significantly induce the expression of some immunoactive molecules, such as C3 and CXCL6, and to downregulate the expression of others including CCL2, CCL25, CXCL14, IL10, or IL13. It should be noted, however, that in some cases such as IL10 the magnitude of the change in gene expression, although significant, was small (less than 2) and perhaps of limited biological relevance. The effect of probiotics, mainly* Lactobacillus *strains, on transcriptional responses of human epithelial cells has been previously assessed both* in vitro* [14–16] and* in vivo* [17–19]. Although the studies on bifidobacteria are scarcer there are also some examples [12, 13, 20, 21]. These studies show a limited response of human intestinal epithelia cells lines to stimulation with bifidobacteria. Nevertheless, it is still interesting to see that our results, although* in vitro*, suggest an effect of the strain* B. breve *IPLA20004 in a number of genes coding for cytokines, chemokines, and receptors, which is in agreement with some* in vivo* studies on the effect of probiotic lactobacilli upon gene expression patterns in the human small bowel [17] and supports a link between the interaction of bacteria with epithelial cells and the immune system. Interestingly, in spite of the different models used, some of the genes found to be modulated in this study have been previously reported to be modulated by probiotic* Lactobacillus *strains both* in vitro* using epithelial cells [22] and* in vivo* in the human small bowel mucosa [17, 19]. To this regard, the colonic epithelial cell line used in our study (HT29) may better resemble the small bowel, where the mucus layer is thin, than the colon where a thick mucus layer is known to be present which prevents the close contact of bacteria with the epithelial cell [9]. Moreover, coculture of mice primary colonic epithelial cells with* L. rhamnosus* GG induced the expression of IL1*β*, TNF*α*, CXCL5 (ENA-78), CXCL10 (IP10), CCL20 (MIP3*α*), CCL2 (MCP1), CCL7 (MCP3), CXCL2 (MIP2*α*), and CCL5 (RANTES) [22], and our results indicated a significant downregulation of CCL2 without affecting the other* L. rhamnosus* GG-induced genes. This may suggest a differential response to our bifidobacteria with regard to* L. rhamnosus* GG, although the influence of the different colonocyte models used cannot be overruled. Administration of* L. rhamnosus* GG to human volunteers induced the expression of some of these genes (CCL24, CCL2, CXCL3, CXCL13, CXCL12, CCR3, CCL19, CCL21, or lymphotoxin-*β* [LTB], among others) on the small bowel mucosa, whilst other* Lactobacillus* strains (*L. acidophilus* Lafti L10) resulted in a different expression profile (inducing CXCL10 and CXCL11, among others) [19]. On the contrary, generalizing, in our* in vitro* model* B. breve* IPLA20004 tended either to downregulate or not to affect these genes which suggest a limited stimulatory activity of this strain when compared with the immune-stimulatory ability of lactobacilli. It should be noted, however, that the differences existing between the* in vivo* studies and our* in vitro* results with HT29 cells may be partly related to the different experimental conditions used; for instance, we performed the incubations under a 5% CO_2_ atmosphere in comparison with the anaerobic intestinal environment which may have an effect on an anaerobic microorganism such as* B. breve*.

As indicated above, chemokines function mainly as chemoattractants for leukocytes, recruiting monocytes, neutrophils, and other effectors cells from the blood to sites of infection or tissue damage [23]. Chemokines such as CCL2 or CCL25 attract immune cells, such as macrophages and T-lymphocytes expressing their receptors (CCR2 and CCR9, resp.) to the tissue [23]. Actually, expression of CCR9 has been found to be involved in the homing to the intestine of thymic T-cells [24]. On the other hand, the only chemokine gene found to be upregulated in our study was that of CXCL6 (human granulocyte chemotactic protein-2, GCP-2). This chemokine attracts and activates neutrophils [25] being, together with IL8, the only CXC-family chemokine recognized by both CXCR1 and CXCR2 receptors. IL8 is the most active protein chemoattracting neutrophil, although in our study its gene was not found to be significantly upregulated and IL8 determination in the supernatants showed higher, but not statistically significant, values. Moreover, a recent study demonstrated an increased production of the chemokine CXCL16 (not studied in this work) in germ-free animals, which resulted in an increased recruitment of immune cells to the intestinal mucosa [26]. This underlines the importance of chemokines in immune cells recruitment and the modulation of their production by the intestinal microbiota.

Interestingly, the complement component C3 was among the most strongly upregulated genes in the small bowel mucosa after administration of* L. rhamnosus* GG to healthy volunteers [17]. Similarly, the expression of this gene in HT29 cells was the most clearly upregulated by our* B. breve* strain and a significantly higher production of C3 by the epithelial cell line was confirmed by means of ELISA tests. C3 is the most abundant complement protein in serum, it enhances phagocytosis promoting innate immunity, and it is also important for an effective antibody response, thus constituting a link between the complement system and the acquired immune response [27]. In our study the downregulation of the expression of genes such as CCL2 or CCL25 together with the upregulation of the expression of CXCL6 and C3 by colonic cells suggests a local effect by suppressing the recruitment to the mucosa of lymphocytes and by increasing that of the innate immunity cells such as neutrophils and mastocytes. However, the limitations of our study design do not allow the establishment of firm conclusions on whether the differences obtained with regard to the reports by other authors are due to the different strains used or to the models' responsiveness.

Finally, in order to complement the data on the interaction between* B. breve* IPLA20004 and HT29 cells we performed a proteomic approach. This analysis allowed us to detect the overproduction of cytokeratin-8 (CK-8) or type I cytoskeletal 8, a keratin protein encoded by the krt8 gene; this protein is located in the nucleoplasm and the cytoplasm where, as a part of the cytoskeleton, it is known to help to link the contractile machinery to dystrophin at the costamere in striated muscle cells [28]. Interestingly, this strain has been previously found to increase the transepithelial resistance of the HT29 cell monolayer [13] which may be correlated with this induction of changes in the cytoskeleton. Moreover, the chain A of the tapasin-ERp57 was also overproduced. The heterodimer formed by tapasin-ERp57, linked by a stable disulfide bond, is part of the major histocompatibility complex (MHC) class I peptide-loading complex [29]. This heterodimer has been shown as the functional unit for loading MHC class I molecules with high-affinity peptides [30]. It has been shown that upregulation of tapasin may facilitate optimal peptide loading on the MHC class I molecule [31], although the putative functions in enterocytes have passed unnoticed until now.

In this study we have determined the effects of* B. breve* IPLA20004 on intestinal epithelial cells, observing a potential improvement of the epithelial barrier. This, together with previous studies carried out on the interaction of the strain with immune cells indicating a Th1 profile [8, 12] or showing an increase of the transepithelial resistance of the colonic epithelial cells monolayer [13], suggests the interest in conducting experiments in which both polarized epithelial cells and immune cells are cocultured.

In summary, our results suggest that this strain offers possibilities for increasing the intestinal barrier against pathogens in populations in which the barrier may be compromised. This could be achieved by two independent mechanisms: firstly by strengthening the cell cytoskeleton and, therefore, the physical resistance of the epithelial layer and secondly by modulating the immune environment at local mucosal level towards a “prestimulated” innate immune response by recruiting immune cells.

## Supplementary Material

Supplementary Figure 1. Proteomes of HT29 cells co-cultured with or without B. breve IPLA20004. Arrows indicate the two proteins significantly (p<0.05) up-regulated in the HT29 cells by the B. breve strain. 1- cytokeratin 8 and 2- chain A of the tapasin-ERP57.

## Figures and Tables

**Table 1:** Changes in cytokines and receptors gene expression in HT29 cells after exposition to bifidobacteria when compared to exposition to culture medium without bifidobacteria (control), as determined by RT-PCR.

Up- or downregulation (compared to control)		
Gene	*B. breve* IPLA20004	
	Fold regulation	*P*
C3	17.71	*0.001 *
CCL2	−3.29	*0.026 *
CCL25	−5.32	*0.039 *
CCR1	−2.60	*0.012 *
CCR4	−6.07	*0.006 *
CCR5	−10.36	*0.004 *
CCR7	3.19	*0.011 *
CXCL6	2.12	*0.028 *
CXCL14	−2.43	*0.015 *
IL10	−1.64	*0.021 *
IL13	−2.75	*0.009 *
XCR1	−3.65	*0.001 *

**Table 2:** Effect of *B*. *breve* IPLA20004 on cytokines and C3a levels in HT29 cells supernatants. All the results are expressed as pg/mL. Control cells were exposed to culture medium without bifidobacteria.

	Concentration (pg/mL)		
	Control	*B. breve* IPLA20004	*P*
IL10	0.43 ± 0.44	0.83 ± 0.38	0.302
IL12p70	0.19 ± 0.03	0.30 ± 0.02	*0.007 *
IL1*β*	0.17 ± 0.17	0.26 ± 0.20	0.604
TNF*α*	2.19 ± 1.45	3.35 ± 0.18	0.242
IL8	264.82 ± 38.41	423.51 ± 200.29	0.249
C3a	217.33 ± 38.37	311 ± 42.14	*0.045 *

## References

[1] Food and Health Agricultural Organization of the United Nations and World Health Organization (2002). Guidelines for the evaluation of probiotics in food. *Working Group Report*.

[2] Yatsunenko T., Rey F. E., Manary M. J. (2012). Human gut microbiome viewed across age and geography. *Nature*.

[3] Collado M. C., Gueimonde M., Pérez-Martínez G. (2011). Current and future applications of probiotics. *Current Nutrition and Food Science*.

[4] Gareau M. G., Sherman P. M., Walker W. A. (2010). Probiotics and the gut microbiota in intestinal health and disease.. *Nature reviews. Gastroenterology & hepatology*.

[5] Binns N. (2013). *Probiotics, Prebiotics and the Gut Microbiota*.

[6] Maynard C. L., Elson C. O., Hatton R. D., Weaver C. T. (2012). Reciprocal interactions of the intestinal microbiota and immune system. *Nature*.

[7] Christensen H. R., Frøkiær H., Pestka J. J. (2002). Lactobacilli differentially modulate expression of cytokines and maturation surface markers in murine dendritic cells. *The Journal of Immunology*.

[8] López P., Gueimonde M., Margolles A., Suárez A. (2010). Distinct *Bifidobacterium* strains drive different immune responses in vitro. *International Journal of Food Microbiology*.

[9] Swidsinski A., Loening-Baucke V., Lochs H., Hale L. P. (2005). Spatial organization of bacterial flora in normal and inflamed intestine: a fluorescence in situ hybridization study in mice. *World Journal of Gastroenterology*.

[10] Brandtzaeg P. (2011). The gut as communicator between environment and host: Immunological consequences. *European Journal of Pharmacology*.

[11] Arboleya S., Ruas-Madiedo P., Margolles A. (2011). Characterization and in vitro properties of potentially probiotic *Bifidobacterium* strains isolated from breast-milk. *International Journal of Food Microbiology*.

[12] López P., González-Rodríguez I., Gueimonde M., Margolles A., Suárez A. (2011). Immune response to *Bifidobacterium bifidum* strains support Treg/Th17 plasticity. *PLoS ONE*.

[13] López P., González-Rodríguez I., Sánchez B. (2012). Interaction of Bifidobacterium bifidum LMG13195 with HT29 Cells Influences regulatory-T-cell-associated chemokine receptor expression. *Applied and Environmental Microbiology*.

[14] Mack D. R., Ahrne S., Hyde L., Wei S., Hollingsworth M. A. (2003). Extracellular MUC3 mucin secretion follows adherence of *Lactobacillus* strains to intestinal epithelial cells in vitro. *Gut*.

[15] Schlee M., Harder J., Köten B., Stange E. F., Wehkamp J., Fellermann K. (2008). Probiotic lactobacilli and VSL#3 induce enterocyte *β*-defensin 2. *Clinical and Experimental Immunology*.

[16] Paolillo R., Carratelli C. R., Sorrentino S., Mazzola N., Rizzo A. (2009). Immunomodulatory effects of *Lactobacillus plantarum* on human colon cancer cells. *International Immunopharmacology*.

[17] di Caro S., Tao H., Grillo A. (2005). Effects of *Lactobacillus* GG on genes expression pattern in small bowel mucosa. *Digestive and Liver Disease*.

[18] Troost F. J., Van Baarlen P., Lindsey P. (2008). Identification of the transcriptional response of human intestinal mucosa to *Lactobacillus plantarum* WCFS1 in vivo. *BMC Genomics*.

[19] Van Baarlen P., Troost F., van der Meer C. (2011). Human mucosal in vivo transcriptome responses to three lactobacilli indicate how probiotics may modulate human cellular pathways. *Proceedings of the National Academy of Sciences of the United States of America*.

[20] Riedel C. U., Foata F., Goldstein D. R., Blum S., Eikmanns B. J. (2006). Interaction of bifidobacteria with Caco-2 cells-adhesion and impact on expression profiles. *International Journal of Food Microbiology*.

[21] Shima T., Fukushima K., Setoyama H. (2008). Differential effects of two probiotic strains with different bacteriological properties on intestinal gene expression, with special reference to indigenous bacteria. *FEMS Immunology and Medical Microbiology*.

[22] Lan J.-G., Cruickshank S. M., Singh J. C. I. (2005). Different cytokine response of primary colonic epithelial cells to commensal bacteria. *World Journal of Gastroenterology*.

[23] Niess J. H., Reinecker H. C. (2005). Chemokines in immune surveillance of the intestine. *Current Topics in Membranes*.

[24] Cebula A., Seweryn M., Rempala G. A. (2013). Thymus-derived regulatory T cells contribute to tolerance to commensal microbiota. *Nature*.

[25] Wuyts A., Struyf S., Gijsbers K. (2003). The CXC chemokine GCP-2/CXCL6 is predominantly induced in mesenchymal cells by interleukin-1*β* and is down-regulated by interferon-*γ*: comparison with interleukin-8/CXCL8. *Laboratory Investigation*.

[26] Olszak T., An D., Zeissig S. (2012). Microbial exposure during early life has persistent effects on natural killer T cell function. *Science*.

[27] Sahu A., Lambris J. D. (2001). Structure and biology of complement protein C3, a connecting link between innate and acquired immunity. *Immunological Reviews*.

[28] Stone M. R., O'Neill A., Catino D., Bloch R. J. (2005). Specific interaction of the actin-binding domain of dystrophin with intermediate filaments containing keratin 19. *Molecular Biology of the Cell*.

[29] Wearsch P. A., Cresswell P. (2013). *In vitro* reconstitution of the MHC class I peptide-loading complex. *Methods in Molecular Biology*.

[30] Wearsch P. A., Cresswell P. (2007). Selective loading of high-affinity peptides onto major histocompatibility complex class I molecules by the tapasin-ERp57 heterodimer. *Nature Immunology*.

[31] Kaneno R., Shurin G. V., Kaneno F. M., Naiditch H., Luo J., Shurin M. R. (2011). Chemotherapeutic agents in low noncytotoxic concentrations increase immunogenicity of human colon cancer cells. *Cellular Oncology*.

