# Supplementary figures and images for: The Effects of Bifidobacterium breve on Immune Mediators and Proteome of HT29 Cells Monolayers
Source: Biomed Res Int. 2015 Feb 22;2015:479140. doi: 10.1155/2015/479140 (PMC4352474; doi:10.1155/2015/479140)

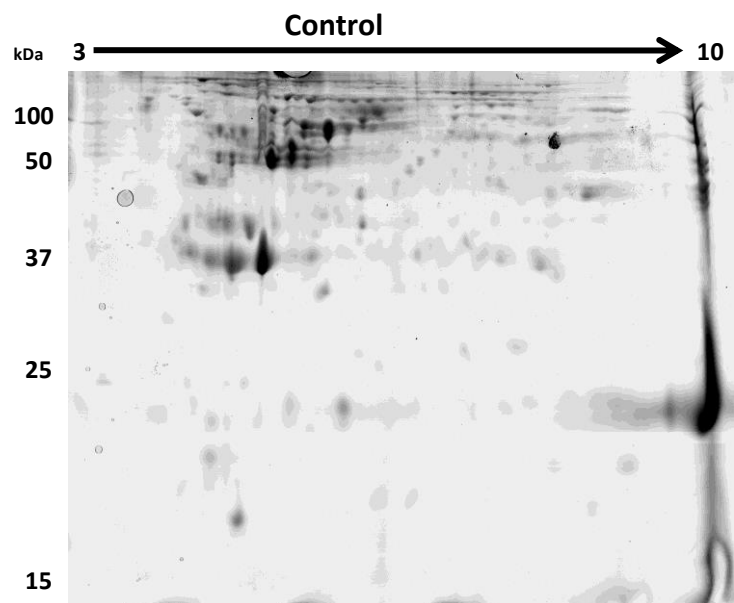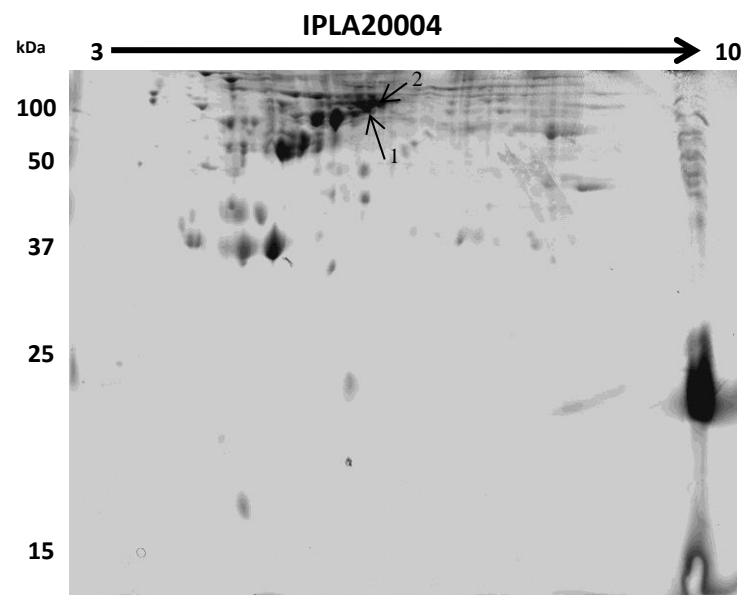

Supplement: Supplementary file 1 — Supplementary Figure 1. Proteomes of HT29 cells co-cultured with or without B. breve IPLA20004. Arrows indicate the two proteins significantly (p<0.05) up-regulated in the HT29 cells by the B. breve strain. 1- cytokeratin 8 and 2- chain A of the tapasin-ERP57. [file 479140.f1.pdf]
